# AI-Enabled Opportunities and Transformation Challenges for SMEs in the Post-pandemic Era: A Review and Research Agenda

**DOI:** 10.3389/fpubh.2022.885067

**Published:** 2022-04-29

**Authors:** Xiaoqian Lu, Kumud Wijayaratna, Yufei Huang, Aimei Qiu

**Affiliations:** ^1^School of Business Administration, Jimei University, Xiamen, China; ^2^Newcastle Business School, Northumbria University, Newcastle Upon Tyne, United Kingdom; ^3^University of York Management School, University of York, York, United Kingdom; ^4^School of Business, Guangdong University of Foreign Studies, Guangzhou, China

**Keywords:** post-pandemic recovery, pandemic, AI technology, SMEs, continuous development

## Abstract

The negative impact of COVID-19 pandemic has seen SME's struggling around the world. With many quickly adopting digital technologies, such as AI, in their manufacturing or services operations to achieve sustainable development. This study aims to develop a framework that informs AI-enabled sustainable development for SMEs by integrating the relevant research in the field. In this framework, we identify the opportunities that the deployment of AI technology can do to alleviate the plights of SMEs in the post-pandemic era, including the impacts on work, organizations, and performance. We further explore the challenges that SMEs face in AI transformation and recommend strategies to take on those challenges. Finally we propose an agenda for future research based on technological challenges and environmental threats.

## Introduction

Small and medium-sized enterprises (SMEs) are the main driving force of employment and economic growth (1). The outbreak of COVID-19 in 2020 and resultant (regional) pandemic control and lockdown policies have made SMEs vulnerable (2). Compared with large corporations, SMEs are facing greater challenges and uncertainties (3, 4), which will likely continue to the post-pandemic era (5, 6). Despite the rapid response of governments to curb business closures by providing mitigation measures such as loans, wage support and subsides, the financial difficulties of SMEs have not been significantly improved. Some mitigation measures to reduce this burden are not as effective as expected, therefore its vital to find urgent measures to properly resolve the difficulties of SMEs and subsequently promote their continuous development.

The surge of digital technology has triggered innovations in products, services, processes (7), and business models (8, 9). Artificial intelligence (AI) plays a leading role in this transformation. AI is usually described as an advanced digital form (10), and can be defined as an advanced prediction technology (11), including but not limited to machine learning (ML) and deep learning, natural language processing (NLP) and machine vision (12, 13). During the COVID-19 pandemic, the use of AI technology has been an essential solution to maintain the sustainability of SMEs (14). The adoption of AI technology can help SMEs turn COVID-19 challenges into opportunities to improve performance and improve their survival rate (4). The extant studies mainly focus on the predicaments experienced by SMEs and how organizations participants interact during the crisis (4, 15–18). Although there is growing research interest on the role of AI technology in SMEs' survival and development during the crisis (4, 14, 19), there is a lack of conceptual framework for understanding AI-enabled sustainable development of SMEs and guiding future research in the post-pandemic era.

This study aims to develop a conceptual framework that informs AI-enabled sustainable development for SMEs by integrating the relevant research in the field. This is achieved by specifically reviewing most recent literature and attempting to answer the following research questions. First, what can the deployment of AI technology do to alleviate the plights of SMEs in the post-pandemic era? For example, where can AI be deployed? What are the benefits? What are the impacts on work, organizations, and performance? Secondly, what kind of challenges do SMEs face in AI transformation (or deployment)? How do we solve these challenges? Which stakeholders need to work together and in which direction?

To address the above questions, we focus on reviewing the relevant research outputs published since 2019, when the COVID-19 pandemic started. The next section details the methodology adopted. We integrate the main theoretical viewpoints, themes of actions or strategies that SMEs should consider when developing sustainability by using AI technology. Next, we explore the challenges of AI transformation of SMEs and proposes future research directions based on the technological challenges and environmental threats.

## Methodology

**Figure 1:**
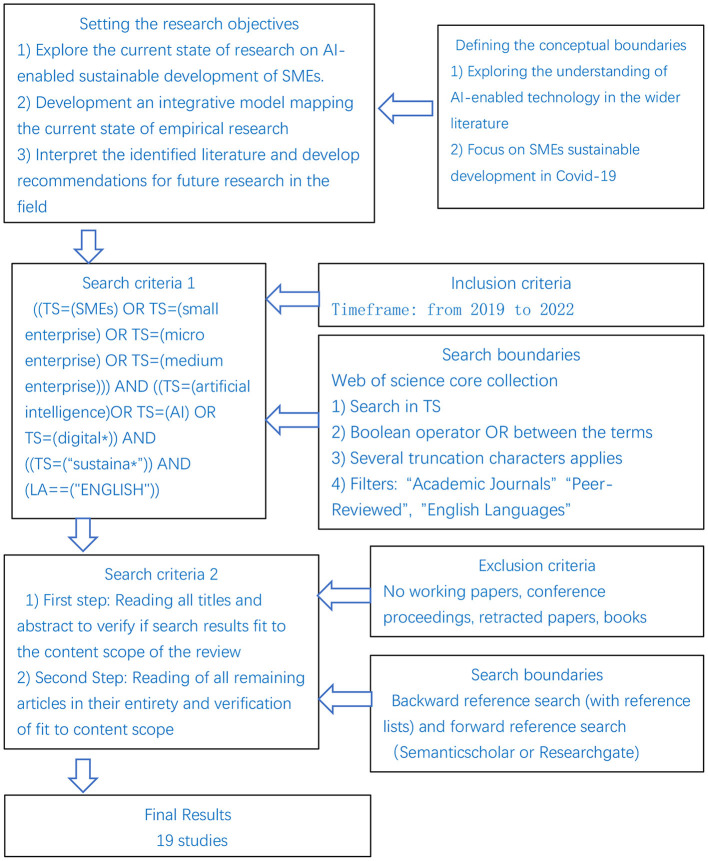
Literature review process.

This study adopts the realist systematic approach to reviewing the relevant literature (20, 21). The detailed search process is shown in Figure 1. We first determined the most basic search terms through informal search and applied them to the core collection of Web of Science, and then expanded the collection scope according to the citation of the literature. Specifically, under the TS column of Web of Science, we first use small and medium-sized enterprises (e.g., “SMEs,” “small enterprise,” “micro enterprise,” and “medium enterprise”), artificial intelligence or digitization (e.g., “artistic intelligence,” “Ai,” and “digital ^*^”) and sustainable development (e.g., “sustaina ^*^”) as keywords. The timeframe is limited to “2019–2022”, The literature type is limited to “articles, Early access or review”, and 62 articles are obtained through preliminary search. Next, we exported the detailed information of these studies [i.e., journal, author, title, year, abstract, and PDF (if any)] to an Excel spreadsheet.

We then checked whether these articles met the scope of the review and further refined the keywords by browsing the titles, abstracts, and keywords. After eliminating irrelevant and duplicate studies, we retained 10 articles. Next, according to these articles, we carried out a pre-citation and post-citation search (22), and expanded the search scope to electronic databases such as Springer and ScienceDirect. Using this method, we finally obtained 19 articles. Collectively these articles explain the mode of AI technology enabling SMEs to promote their sustainable production and consumption.

## Findings

**Figure 2:**
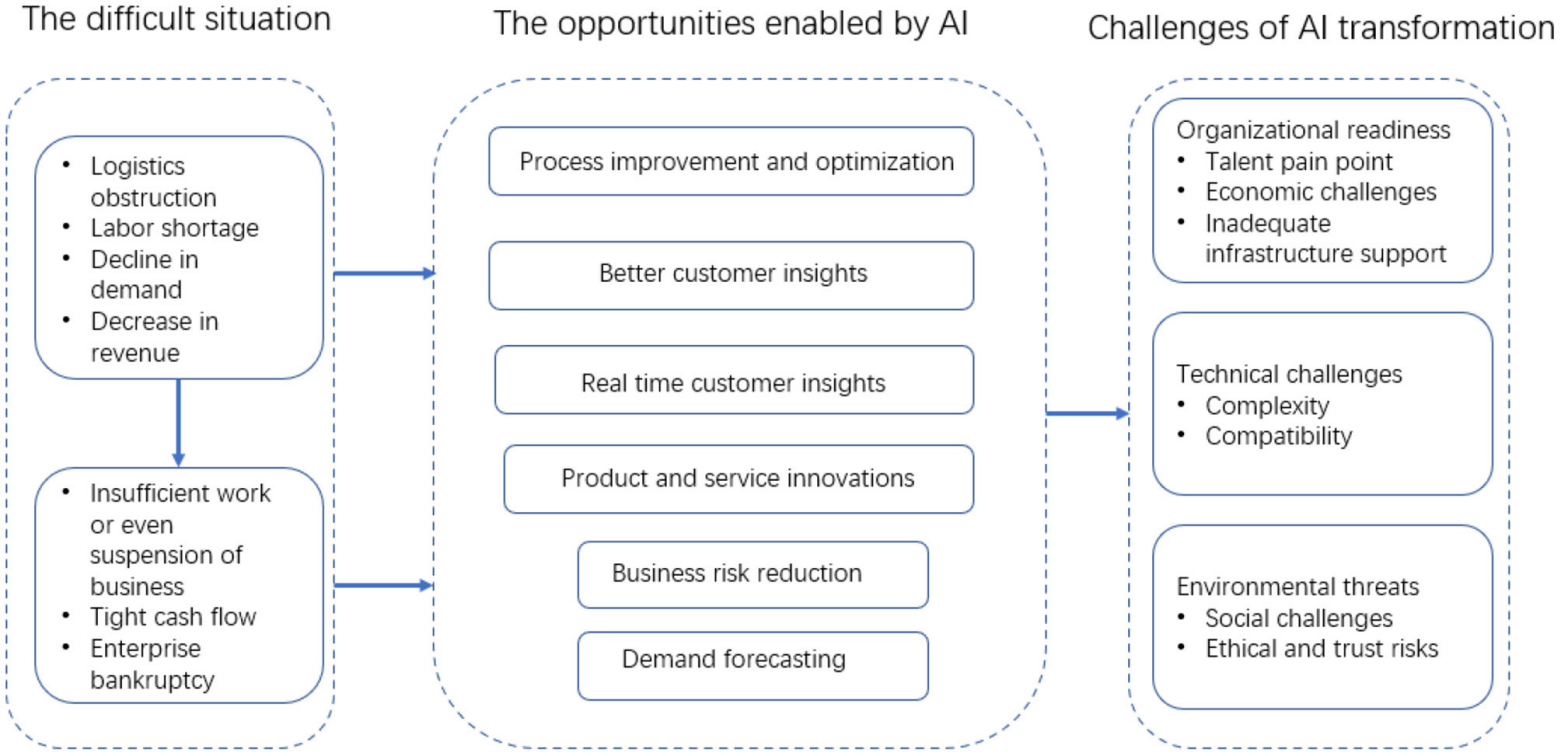
AI-enabled opportunities and transformation challenges for SMEs.

The findings of our review are organized in a framework as presented in Figure 2. We first provide an overview of the challenging environment faced by SMEs during the COVID-19 pandemic, followed by a discussion of the opportunities provided by AI technologies, and the challenges of AI transformation.

### The Difficult Situation Brought by the Pandemic

The COVID-19 pandemic and the lockdown and other control measures have made SMEs' operating environment the most fragile and unpredictable ever in recent history (2, 4). First, the destroyed global production and business practices have increased the uncertainty of the production and operation of SMEs (23). Second, the global or regional logistics is blocked and the supply chain is interrupted (4). Many raw materials urgently needed for production are difficult to be in place in time. In addition, due to the shortage of labor, the decline of demand, and the cancellation of orders, a large number of manufacturing SMEs have ceased to operate (24). Third, interaction or working time restrictions and the decline of customers' purchasing power reduced or suspended business activities, and the business revenue shrank sharply. The cash flow shortage of SMEs (especially tourism, catering, and other service industries) with small capital reserves was even more obvious. This change in the business environment occurred during the pandemic but will last for a long time after the pandemic (5, 6).

### Opportunities Enabled by AI

New technologies such as AI can help SMEs win competitive advantages or provide means of survival in the fields of manufacturing, e-commerce, accounting, human resources, marketing, and customer relations (25). Research of AI technology enabling SMEs during the pandemic mainly focuses on two aspects: One is to improve the operation of SMEs through gradual process improvement and optimization within the organizational boundary; the other is the external AI-driven transformation to fundamentally promote SMEs to create business models, develop new organizational strategies and cultures, build business alliances, and others (3).

#### Process Improvement and Optimization

Solutions based on AI can help manufacturing SMEs realize automation, which leads to process improvement and optimization (11). AI can help to solve the problem of labor shortage and enable SMEs to achieve world-class production standards and promotion activities with the least manpower (26). It can automate customer management, collect and process advanced data to improve trend analysis, logistics planning and inventory management, reduce costs, increase sales and increase profits. It can save time and limit defects, avoid risks and improve performance (12), so that suppliers can create value closer to customer operations (10). During the pandemic, AI technology provided new service modes for SMEs and realized asymptotic process improvement with the help of image recognition and speech recognition.

#### Real-Time Customer Insights

AI are not only able to identify, verify and monitor customers in real time, but also determine purchasing power and interest in purchased goods, according to use traces, and then market segment products according to customers' specific needs. The pandemic control policy enables most consumers to switch to online consumption, and every customer must leave traces when using online services. Popular online sales provide AI with a large database to understand the actual needs and preferences of customers, and to advise manufacturers or retailers to target specific audiences for advertising, promotion, and sales optimization (27). AI can analyze all activities performed by consumers online, study their behavior and computing possibilities through algorithms, manage demand-supply and perform all back-end operations. AI can also assist in social media marketing and identify new market opportunities. Big data analysis in AI technology can be used to develop customers' personalized files and predict their purchase habits (11), which helps to increase the customer base and overall profitability of SMEs (28).

#### Product and Service Innovations

AI-based innovations such as virtual mirrors and visual search can provide services for garment or tourism organizations to improve customer interaction and narrow the gap between physical and virtual shopping experiences. The future smart kitchen and smart restaurant based on intelligent technology and highly intelligent auxiliary robots can provide a healthier kitchen lifestyle and develop a large health industry. During the COVID-19 pandemic, smart technologies have been widely used in the service industry, enabling the service organizations to carry out business activities within a safe distance, enhance customer delivery, create businesses for the organization, and provide competitive advantages during the new crown pneumonia pandemic. The customer service function of AI (such as chatbot) can help organizations answer simple requests from customers (11).

#### Business Risk Reduction

Business risk is an important factor that hinders SMEs from achieving the goal of sustainable development. Because risk factors have the characteristics of data uncertainty and diversity, cognitive solutions based on AI can supplement the shortcomings of traditional solutions in dealing with complex data. It can solve the situation of fuzziness and uncertainty especially when the data is large or in different dimensions. Algorithms and technical solutions based on AI can help SMEs avoid business risks (7) and move toward sustainable development (29).

#### Demand Forecasting

Due to the difficulty of demand forecasting caused by the pandemic, it is more important than ever to understand and predict consumer demand through an integrated supply chain, and AI technology may become a key component (11). In retail SMEs can use AI technology in demand forecasting and supply chain implementation, thereby informing the ability to trigger response demand when customers choose the last item and then track the store's rapid replenishment of products (11). SME manufacturing firms can fight against tacit knowledge by using AI to promote technologies, such as decision support systems (DSS) (12, 30). The Internet of things combined with AI can also be used for predictive analysis in maintenance to reduce machine downtime and increase part-production (12).

The COVID-19 pandemic has driven SMEs to open the upstream and downstream ecology of the industry, fully meet the broad market demand of AI applications, and realize a complete industrial closed loop. For example, under the lockdown policy during the pandemic, some SMEs in the food supply chain, such as restaurants, cafés and retail organizations, could quickly carry out remote operations and allow online ordering, delivery and distribution (3). Furthermore, by enabling the traditional QR code with AI, the data of various activities (such as cinemas, scenic spots, retail stores, etc.), other than inventory management, can be recorded or executed (27). It can record high-quality data while reducing personnel contact, to understand customer preferences, predict future needs, and provide high-quality data for organizations to improve production efficiency.

#### Business Model Innovations

The potential advantages of AI to develop multiple business models that provide customers with numerous advantages can be realized with AI-driven business models (31), by reducing costs, improving service quality, coordination, productivity, and improving delivery efficiency (7, 11, 32, 33). Advances in AI have enabled organizations to shift from a product-centric model to a more advanced (i.e., platform or result-based) digital business model with higher value creation potential (10, 34, 35).

A core proposition of digital service is that digital technologies such as AI provide fundamental opportunities for services of industrial organizations, create and obtain value from new revenue streams and realize the difference from competitors by assuming greater responsibility to support customer results (10). For example, through investment in AI technology, suppliers can monitor, analyze, and control the performance of automatic connection equipment and provide enhanced digital customer service to realize on-site optimization. AI-enabled automation technology can manage and analyze large amounts of data to recommend action plans and make these decisions (11). For manufacturing organizations, they can focus on agile customer co-creation, data-driven payment operation and scalable ecosystem integration to innovate their business model, and expand AI through business model innovation such as mechanism and feedback cycle (36).

SMEs can take AI as the core and big data as the basis, whilst developing AI capabilities (such as value creation, value delivery and value capture) to carry out technological R & D innovation or business model innovation, to create business activities with higher efficiency. First, to obtain and create new sources of income for SMEs, AI's value capture ability is used. It reduces errors in cost structure, potential income flow, financial feasibility, developing income models, and sorting out cash flow. Furthermore, AI can help SMEs match customer invoices with payments received (11).

Secondly, using AI's value delivery capability, new operational processes and activities can be established to deliver the promised value. For example, AI can help small and medium-sized retail organizations use algorithms to customize products for consumers and analyze data on a large scale in digital marketing, so as to improve efficiency, achieve competition and growth (27).

Thirdly, the value creation ability of AI can be used to help organizations make organizational decisions, obtain information and professional knowledge. For example, algorithms based on AI can find feasible solutions to problems of competitiveness and product quality and help to improve the productivity of SMEs and realize quality optimization and overall development. In the context of digital service aimed at optimizing the use and maintenance of products (product groups) in customer operations, AI services can be used by systematically evaluating AI applications and their potential value to customers and end-users (37). Any value created in digital technology can be created (jointly) in an agile and customizable manner from the perspective of the customer needs (38).

## Challenges of AI Transformation

The COVID-19 pandemic has brought the digital transformation of organizations to the forefront. SMEs need to adopt new technologies to enhance, change or even destroy business models in order to remain competitive (12, 25). Nonetheless, SMEs may lack the necessary strategies, knowledge, and resources to use AI technology (12, 39). Many SMEs (especially manufacturing organizations) are trying to figure out whether AI technology can provide them with answers. Based on Technology, Organization, and Environment (TOE) framework (40, 41), we explore the challenges existing in the AI transformation of SMEs, as shown in Table 1.

**Table 1:** Challenges of AI transformation for SMEs.

**Area**	**Challenges**	**References**
Organization readiness	(1) Manpower: enterprise leaders (managers) lack the knowledge and transformation consciousness of applying AI; Enterprises lack experts in the field of AI; Employees lack the ability of AI	(6, 12, 32, 42–46)
	(2) Financial resources: digital paradox or digital trap - enterprises face economic challenges of high income, high input cost and low actual return.	(8, 37, 43, 45, 47, 48)
	(3) Material resources: poor infrastructure support and data acquisition ability of enterprises.	(11, 27, 42, 49)
Technology challenges	(1) Complexity: practicability, ease of use and application cost.	(1, 27, 47, 50)
	(2) Compatibility: data quantity and quality, transparency, data standard and data structure.	(11, 30, 45)
Environmental threats	(1) Cooperation of employees, customers, and other stakeholders: form a good data use ecosystem.	(9, 10, 36–38, 51)
	(2) Ethics and trust risk: moral problems, trust problems; Legal issues; User privacy issues, etc.	(11, 33, 41, 43)

### Organization Readiness

A considerable number of SMEs fail to successfully integrate AI capabilities into their business models due to the lack of readiness by the organization, which includes the readiness in human, financial, and material resources (42).

#### Human Resources

To seize the opportunities enabled by AI, firms need to develop the capabilities of using AI technologies (11). Lack of AI knowledge and professional skills is the primary challenge for the implementation of AI in most SMEs (12, 26, 52). AI systems do not yet have the essence of human intelligence (53), therefore AI algorithms will not produce clear answers, but will provide tentative solutions (for example, probability-based prediction), which requires human explanation, demonstration, and action to create specific and valuable results (54). The choice of AI level of SMEs may depend on human factors (11), such as organizational leaders, experts, employees and so on.

Successful AI transformation is more important to solve management problems (3), including redesigning business processes, human resources and organizational capabilities. This depends on top management support (27, 40). However, most leaders of SMEs either lack the necessary AI knowledge (12) or the awareness of AI transformation (6), resulting in an insufficient understanding of the potential value and advantages of AI technology (42, 43); or have unrealistic expectations for AI technology (43). The expectations from AI exceed the ability of the organization; or it is difficult to solve the gap between the current and required AI capabilities (44), so it does not support the adoption of AI.

Talent shortage and skill gap are one of the primary strategic challenges of most SMEs in the AI environment (11, 26). At present, many practitioners do not have a clear understanding of the capabilities and basic procedures required by organizations to obtain the potential of AI (32), and they are unable to solve the problem of human resources required for the promotion of AI (42, 43, 45). When employees generally lack the necessary AI knowledge (12), there are few employees training and developing skills necessary for AI application. Consideration needs to be given to how SMEs, especially small organizations, find and retain skilled talents.

#### Financial Resources

The cost of digital technology (25) and financial return are important factors that SMEs need to consider. The application of AI in SMEs is more likely to produce the so-called “digital paradox”, due to the rising cost (3), even the increase of digital service revenue cannot bring greater profits to organizations (8). In the digital economy of the post-pandemic era the development of computing power was limited, not always readily available nor affordable when SMEs consider cost efficiency, even though, utilizing digital services and the implementation of AI can strategically develop and provide numerous sources of new revenue. However, the increasing availability of solutions with advanced functions suggests higher entry investment and maintenance costs are required (55), which results in a rise in organizations service costs (8).

Previous studies have shown that industrial 4.0 technologies such as autonomous robots and network physical systems are not feasible for SMEs because of the high cost (47). Organizations with tight cash flow; the introduction of AI-based technology may affect the profitability and increase the use cost of consumers (43), resulting in SMEs falling into an unavoidable digital trap, whilst not understanding fully the value delivery and AI capture (37).

#### Material Resources

There is little doubt that digital technology has improved quality and efficiency, according to Sjödin et al. (36) it is important to recognize the digitization of SMEs and the need to expand AI in digital services (56). The construction of new infrastructure such as the industrial internet is the key to the digital upgrading transformation of SMEs.

Organizations need a lot of data to adopt or deploy AI (42), which depends on the type of infrastructure provided by enterprises (27), and the data available to them. However, a considerable number of SMEs currently lack the high-quality infrastructure to support the adoption of AI (3). Furthermore, SMEs may hinder the improvement of AI solutions because they are unable to obtain a large amount of data or obtain low-quality data (11).

### Technological Challenges

Fully functional AI requires digital connection, at the core of digitization, there are many supporting technologies enhancing the performance of products and services in a variety of ways. However, digitization may influence different stages of the co-creation process in a complex and unclear way (8, 12, 57, 58). According to innovation diffusion theory (59), the diffusion of AI technology needs to consider five aspects: the comparative advantage, compatibility, complexity, testability, and observability of technology. The relative advantages of technology (such as the financial return on investment mentioned in Section The Difficult Situation Brought by the Pandemic) and the ease of use (complexity) of the product itself (41) along with compatibility are important factors determining the successful adoption of AI (11).

#### Technology Complexity

The concept of technical complexity is conceptualized through ease of use (40). Therefore, Technology Acceptance Theory (TAM) should be considered by SMEs when adopting AI for its practicability (27) and ease of use (40). The adaptation of cloud computing solutions are high in SMEs as its simple and easy to understand, easy to use and practical (47), or ease of use of social media platforms that have been widely used in the development of new products for SMEs (1). Therefore, continuous improvement of one's own processes and systems, further supported by investment in technologies, are the key areas for SMEs to maintain competitiveness (12). Nonetheless, the complexity of AI technologies (such as deep learning) is not acceptable and far too complex for most SMEs.

#### Technology Compatibility

Compatibility is the degree to which technology is assimilated and integrated within an organization's existing processes and available infrastructure (40). Currently, there is a lack of unified standards amongst products, equipment, software, and systems related to AI. The implementation of AI is down to the quality of data; however, AI applications face a variety of challenges in this area. These challenges include an insufficient size of data availability, data collection standards, format, integration, continuity, transparency, repeatability, quantity, quality of data input, and dimensional obstacles (11). The data challenges are more obvious to SMEs, especially standards and data structures. Generally, SMEs lack data, or the standardized formats required to verify limited data to gain a real benefit from AI solutions. Simply AI requires the need to create a consistent format that is able to share data among different organizations or industries (11). However, it is questionable whether different algorithms for an SME can solve problems based on different organizations or industries.

### Environmental Threats

Various environmental threats may become a dilemma for the generalization or expansion of AI services for SMEs.

#### Social Challenges

The successful adoption of AI technology requires the cooperation of many stakeholders. Thereby creating a digital service-oriented business model of organizations with a high-quality data ecosystem. However, bringing stakeholders together can be challenging, a shared ecosystem of digitization can affect the business model of any single organization, therefore, requires the business models of other organizations to be consistent (10). In an AI innovation business model, the AI capabilities are assimilated into business activities that create and deliver value to capture and ensure scalable growth (36). This innovative process requires expanding AI services from initial proof of concept through to a larger customer base using AI business models that demonstrate the value of the products, so as to jointly create customer interactions (38). From a value delivery perspective, an organization must act as an entity cooperating with suppliers to adopt and take advantage of emerging opportunities of AI function discovery (9, 38, 51). However, many organizations fail to fully consider the dimension of value delivery (37).

Successful transformation of AI within SMEs needs the support of the government and other organizations. The government can play a key role in supporting the SMEs, by improving the existing legal and regulatory systems, raising the awareness of digital transformation, providing technical and financial support, strengthening data communication infrastructure (3, 45). There is an urgent need in terms of technology and data communication infrastructure for the government to intervene and provide financial support. Equally, due to the wide variety of technical and financial support required, it may become a challenge from a policy point of view.

#### Ethical and Trust Risks

The collection of large data sets, its storage and sharing of that data derived from AI technology and raises ethical issues related to governance, quality, security, standards, privacy, and data ownership (60). Therefore, SMEs need to take certain measures to reduce these risks when using AI. For example, trust is an important factor in the diffusion of AI technology (11, 41). Individuals and organizations may lack trust and worry about the moral dimension of the AI systems and the use of shared data (43). This is prevalent when there are analyses before decision-making based on the AI system. However, it is not clear how to solve moral and legal problems (11, 33). Developing solutions based on AI requires a large amount of data, creating an element of risk due to user privacy, especially when the government issues user privacy laws that propose data collection cannot be at the expense of user privacy. The data collection based on protecting user privacy may limit the development of AI within SMEs, particularly “specialized and new” organizations who would worry about potential risks such as core technology leakage caused by data sharing.

## An Agenda for Future Research

According to Fletcher and Griffiths (5), the COVID-19 pandemic has increased the digital transformation of all organizations. Countries all over the world and in particular China have proposed solutions for the digitization and intellectualization of SMEs. However, it must be noted relevant research on the application of AI technology in SMEs is still very limited. Under continuous optimization of business environment and guidance of national strategic emerging industry policies, it is possible to focus further on organizational, technical, and environmental challenges faced by SMEs when adopting AI technology. The feasibility, applicability, and promotion possibility of AI enabling SMEs are explored further in Table 2.

**Table 2:** Future research directions of AI transformation of SMEs.

**Title**	**Future research directions**
Organization readiness	• What are the key success factors affecting the AI transformation strategy of SMEs? • To implement and use AI on a large scale in response to the pandemic, what special skills, resources and knowledge do SMEs need to realize the transformation strategy of AI? • What challenges may SMEs face in participating in AI transformation in response to the pandemic? • How to change the operation mode to increase the scale, scope and learning opportunities of AI in the organization? • How to select AI automation tasks? How automated is each task? • How can human intelligence provide support in the case of automation errors in AI? • How to keep the infrastructure related to AI technology of SMEs always available? What are the appropriate systems and support personnel needed? • How can supply chain finance help SMEs use the potential of AI technology to obtain financial services? • How can digital payment help SMEs integrate into digital transformation?
Technology challenges	• Understand whether SMEs should adopt AI strategies adopted by large organizations or formulate new AI strategies? How to develop and apply simple processes, systems, and technologies for SMEs? • How to use the data from machines in different SMEs to enable operators and managers to investigate accidents and make fact-based decisions? • How to ensure better quality control and production monitoring by implementing powerful machine learning methods such as machine vision, or at least as auxiliary tools? • To expand the ability of SMEs in monitoring machinery and scheduling processes, what optimization algorithms are there to enable them to quickly produce cheaper monitoring results? • What are the specific methods of applying AI to normative analysis in small and medium-sized manufacturing organizations? • Focusing on specific scenarios, this paper discusses what human capabilities can be enhanced by AI from the perspective of building cost-effective AI solutions?
Environmental threats	• What types, scope and strength of support can the government and relevant industry organizations provide? • What specific policies, regulations, ethical guidance, and legal frameworks need to be formulated and implemented by government regulators to promote the intelligence of SMEs? • What are the criteria for the appropriateness of the policies, regulations, ethical guidance, and legal framework developed and implemented by government regulators?
	• What are the cultural and socio-economic challenges to the AI technology acceptance behavior of SMEs? • How does AI technology change the working mode of SMEs? • What are the effects of AI technology changing the working mode of SMEs on users and data privacy? • What specific policies need to be formulated by SMEs in collecting, sharing, and analyzing data? • How to meet the needs of AI application by collecting the absolute minimum data with the consent of the subjects?

### Future Research on Organization Readiness

The pandemic has affected organizations in all industries. The current research focuses on the application of AI technology in SMEs in the manufacturing industry and mainly focuses on the optimization, improvement, and upgrading processes. There is limited research in other industries and provides very little in way of new models, therefore, further research in AI technology in SMEs for other industries in the future is necessary and evaluate the preparation of SMEs for AI applications (11).

First, organizations obtaining quality talents is a primary objective. Organizations should focus on training, recruiting, and retaining employees with AI technology (26). Consideration should be given to the appropriate systems that ensure the infrastructure related to AI technology in SMEs is always available to support personnel needs.

Second, understand the principles required to use AI in the heart of the business model, such as what special resources and knowledge are needed by SMEs to formulate the transformation strategy of AI? When multiple organizations participate what are the routines that provide the basis for the large-scale implementation and use of AI in response to the pandemic? What is the accumulated knowledge and what are the interdependent actions (36)? How to change the operation mode to increase the scale, scope, and learning opportunities of AI in the organization (7)? Consider how to integrate AI technology into its Revitalization Strategy in the post-pandemic era to ensure business continuity. At the same time, we should also consider how to apply AI technology to replace/increase additional sales channels to increase the revenue of SMEs.

Thirdly, we should think about how SMEs choose AI automation tasks and the degree of automation of each task (such as information acquisition, information analysis, decision-making, action selection and action implementation). At the same time, we should consider how human intelligence can provide support in case of AI automation errors (11).

Fourthly, investigate the financial difficulties of SMEs after COVID-19 and understand how supply chain finance uses the potential of AI technology to provide financial services to alleviate the dilemma of cash flow reduction and capital interruption (61), thereby helping SMEs to resolve financial pressure when deploying AI technology.

### Future Research on Technology Challenges

Firstly, we should try and understand whether SMEs should adopt the AI strategies implemented by large organizations or formulate new AI strategies (11). Secondly, we should consider how to develop and apply simple processes, systems, and technologies for SMEs.

According to Hansen and Bøgh (12), the future application of AI in SMEs in the manufacturing industry includes descriptive analysis, fault diagnosis analysis, predictive analysis, and normative analysis. Therefore, future research includes: How to use data from machines in different SMEs to enable operators and managers to investigate accidents and make fact-based decisions (12). Secondly, how to ensure better quality control and production monitoring by implementing effective machine learning methods such as machine vision, or at least as auxiliary tools? Thirdly, in order to expand the ability of SMEs in monitoring machinery and scheduling processes, what optimization algorithms enable them to quickly produce cheaper monitoring results (62)? Fourthly, what are the specific methods for manufacturing SMEs to apply AI for normative analysis in the future?

In the event of continuously promoting automation, we must reflect on the concept of human beings in this cycle, the focus of AI is to enhance human ability rather than replace it. Therefore, humans may move upstream of the value chain to focus on design and integration-related activities of machines and manpower as part of the integration of AI (63). Future research should focus on specific scenarios and explore the human capabilities that AI can enhance. How to build cost-effective (simple, easy-to-use, low-cost) and reasonable AI solutions for SMEs that enhance human capabilities.

### Future Research on Environmental Threats

The pandemic may stimulate or accelerate the technological advancements of SMEs, including issues related to data protection and the decision-making process, which may directly affect the activities of the manufacturing sector. At this present moment, SMEs lack resources and knowledge to formulate special transformation strategies (12), and governments or relevant industrial organizations need to provide various support such as awareness enhancement, capacity training, platform construction and scheme simplification (45). Governments enhancing policy measures in helping SMEs obtain big data (64) for example. Furthermore, enabling normal operations to resume and attract new organizations, investors, and talent to the region to build a good AI industrial ecosystem.

Future research can focus on the scope, strength, and type of support the government and relevant organizations can provide. For example developing an AI education strategy that can be embedded in government departments, higher education, and employers (26), whilst developing policies, regulations and ethical guidance and framework (33). This can be formulated and implemented by government regulators in terms of intervention and supervision (7). This will prevent the abuse of AI in the future.

The management of data privacy is the key to the intelligent management of SMEs. It is necessary to explore the impact of security and privacy risks on the application of AI technology in SMEs. First, future research can include paying attention to how AI technology changes the working mode of SMEs, and the impact of this change on users and data privacy. Second, it can consider the specific policies that SMEs need to formulate to collect, share and analyze data (4), so as to determine how to collect the absolute minimum data with the consent of the subjects to meet the needs of AI applications.

## Conclusion

Whether in Europe, the United States or China SMEs have created a number of new employment opportunities and contributed to the economy. To maintain a country's economic stability, the continuous growth of SMEs are paramount. The pandemic and its control measures have created various challenges for the sustainability of SMEs around the world, such as blocked logistics, interrupted supply chain, labor shortages, and reduction in demand. Creating uncertainty and making the most vulnerable SMEs fight for their survival and future development and thereby emphasizing the need for advancement of technology as a possible way forward. This paper integrates the latest research of AI-enabled opportunities and the challenges of AI transformation for SMEs in post-pandemic era. The various opportunities of applying AI to improve the continuous development of SMEs are identified, and the challenges of AI transformation of SMEs are discussed. Further research directions are proposed to help SMEs to better seize the opportunities enabled by AI technologies for continuous development.

## Author Contributions

XL: conceptualization, formal analysis, funding acquisition, and writing—original draft. KW: conceptualization and writing—review and editing. YH: writing—review and editing. AQ: writing—review and editing and project administration. All authors contributed to the article and approved the submitted version.

## Funding

This research was supported by the Major Program of the National Social Science Foundation of China (Grant No. 20VHJ007), Soft Science Project of Fujian Province (Grant Nos. 2020R0066 and 2020R0067), and Xiamen Federation of Industry and Commerce (Grant No. H2021139).

## Conflict of Interest

The authors declare that the research was conducted in the absence of any commercial or financial relationships that could be construed as a potential conflict of interest.

## Publisher's Note

All claims expressed in this article are solely those of the authors and do not necessarily represent those of their affiliated organizations, or those of the publisher, the editors and the reviewers. Any product that may be evaluated in this article, or claim that may be made by its manufacturer, is not guaranteed or endorsed by the publisher.
